# In-Silico Selection of Aptamer Targeting SARS-CoV-2 Spike Protein

**DOI:** 10.3390/ijms23105810

**Published:** 2022-05-22

**Authors:** Yu-Chao Lin, Wen-Yih Chen, En-Te Hwu, Wen-Pin Hu

**Affiliations:** 1 Division of Pulmonary and Critical Care Medicine, Department of Internal Medicine, China Medical University Hospital, Taichung 404333, Taiwan; d10001@mail.cmuh.org.tw; 2 School of Medicine, China Medical University, Taichung 404333, Taiwan; 3 Department of Chemical and Materials Engineering, National Central University, Jhong-Li 32001, Taiwan; wychen@ncu.edu.tw; 4 Department of Health Technology, Technical University of Denmark, 2800 Lyngby, Denmark; etehw@dtu.dk; 5 Department of Bioinformatics and Medical Engineering, Asia University, Taichung 41354, Taiwan; 6 Department of Medical Research, China Medical University Hospital, China Medical University, Taichung 40447, Taiwan

**Keywords:** COVID-19, SARS-CoV-2, molecular dynamics simulation, infectious disease, spike protein, DNA aptamer, aptamer–protein interaction

## Abstract

Aptamers are single-stranded, short DNA or RNA oligonucleotides that can specifically bind to various target molecules. To diagnose the infected cases of severe acute respiratory syndrome coronavirus 2 (SARS-CoV-2) in time, numerous conventional methods are applied for viral detection via the amplification and quantification of DNA or antibodies specific to antigens on the virus. Herein, we generated a large number of mutated aptamer sequences, derived from a known sequence of receptor-binding domain (RBD)-1C aptamer, specific to the RBD of SARS-CoV-2 spike protein (S protein). Structural similarity, molecular docking, and molecular dynamics (MD) were utilized to screen aptamers and characterize the detailed interactions between the selected aptamers and the S protein. We identified two mutated aptamers, namely, RBD-1CM1 and RBD-1CM2, which presented better docking results against the S protein compared with the RBD-1C aptamer. Through the MD simulation, we further confirmed that the RBD-1CM1 aptamer can form the most stable complex with the S protein based on the number of hydrogen bonds formed between the two biomolecules. Based on the experimental data of quartz crystal microbalance (QCM), the RBD-1CM1 aptamer could produce larger signals in mass change and exhibit an improved binding affinity to the S protein. Therefore, the RBD-1CM1 aptamer, which was selected from 1431 mutants, was the best potential candidate for the detection of SARS-CoV-2. The RBD-1CM1 aptamer can be an alternative biological element for the development of SARS-CoV-2 diagnostic testing.

## 1. Introduction

Since the epidemic of the coronavirus disease (COVID-19) began in December 2019, the disease has spread quickly worldwide through person-to-person contact, causing a tremendous number of deaths among humans and economic loss. The official virus name of COVID-19 has been announced by the International Committee on Taxonomy of Viruses for severe acute respiratory syndrome coronavirus 2 (SARS-CoV-2). SARS-CoV-2 is much more infectious than SARS-CoV [1], and it infects the human respiratory epithelial cells through the cell receptor angiotensin-converting enzyme 2 (ACE2), expressed on cell surfaces. The receptor-binding domain (RBD) of SARS-CoV-2 spike protein (S protein) dominates the interaction with ACE2 on the host cell, mediating coronavirus cell entry [2]. The SARS-CoV-2 S protein contains a total number of 1273 amino acids, whereas the RBD in the S1 subunit (319–541 residues) comprises 223 amino acids and is responsible for the ACE2 binding interaction [3]. In addition, the S2 subunit (686–1273 residues) mediates the membrane fusion to help the virus infect the host cell [3,4].

Fast and accurate diagnostic technologies, undergoing necessary quarantine, and applying medical measures to infected patients are important to prevent the spread of the epidemic. The reverse-transcription polymerase chain reaction, which is an accurate and reliable test for the detection of COVID-19, can provide highly specific and quantitative detection of the gene in SARS-CoV-2. COVID-19 antigen rapid test kits were developed based on the lateral flow immunoassay and can be used for the qualitative detection of the SARS-CoV-2 antigen. The sensitivity of rapid antigen tests used for COVID-19 detection is around 85% [5,6]. Most COVID-19 antigen rapid test kits rely on the specific antibody–antigen reaction, and the sample flows through the colloidal gold-labeled antibody in the conjugate layer and then passes through the two test lines (immunoglobulin G (IgG) and IgM) and the control line [7,8]. DNA or RNA aptamers are short and single-stranded (ss) nucleotides (usually with lengths of 12–80 nucleotides) and an alternative to antibodies for immunoassays. Compared with antibodies, aptamers have several advantages, such as robustness, low cost, reusability, ease of chemical modification, low batch-to-batch variability, and low immunogenicity [9,10]. Therefore, aptamers have been utilized in the detection of SARS-CoV-2 S protein using an electrochemical biosensor [11], and the development of aptamer-based lateral flow assays (LFAs) can provide the rapid detection of SARS-CoV-2 to address the COVID-19 pandemic [12].

Two ssDNA aptamers targeting the RBD of the S protein have been recently reported in a study, and they were selected from a large oligonucleotide library via a process called systematic evolution of ligands by exponential enrichment (SELEX) [13]. The interactions between the two DNA aptamers and S protein have also been intensively investigated through protein docking and large-scale molecular dynamics (MD) simulation [14]. In addition to exploring the aptamers against the S protein, the nucleocapsid protein (N protein) is a target for the selection of aptamers that can be used to identify SARS-CoV-2. Chen et al. [15] showed that aptamers designed based on an aptamer for SARS-CoV N protein can still have high-affinity binding for the SARS-CoV-2 N protein, and these aptamers have the potential to be used in antiviral therapy by interfering with the N protein function. Furthermore, other types of aptamers, such as the peptide [16] and quadruplex [17] aptamers, have been designed by in-silico methods for targeting the S protein of SARS-CoV-2.

The application of in-silico methods on the interactions of biomolecules has tremendously promoted the analysis of biological systems, providing valuable information for predicting the activity of biomolecules or understanding biomolecular processes. In-silico approaches are commonly used in drug discovery and development [18,19], protein–protein interaction [20,21], aptamer–protein interaction [22,23,24], etc. Compared with empirical methods, computational in-silico methods have several advantages, such as low cost, a simple process, and queries that can be investigated irrespective of practical limitations that hinder experiments. Therefore, numerous studies have reported the use of in-silico approaches to screen or design aptamers, targeting a variety of biomolecules [16,17,22,25,26,27].

This study aimed to perform the in-silico selection of an aptamer targeting SARS-CoV-2 S protein based on the known sequence of SARS-CoV-2 against aptamers. In our proposed in-silico approach, an aptamer (RBD-1C) reported by Song et al. [13] was used as the parent sequence for the production of one-point and two-point mutated sequences. We adopted this strategy because the production of single, double, and triple-point mutations was evidenced in the microarray experiments, which was a feasible way to optimize and explore the aptamer against a target biomolecule [28]. Aptamers, including the RBD-1C aptamer used in this study, are usually isolated from pools of random-sequence oligonucleotides using affinity-based selection. The binding affinity of the RBD-1C aptamer presents a high affinity to the SARS-CoV-2 S protein, as mentioned in a previous report. However, further refinement of aptamers can achieve better binding affinities. To our knowledge, no report investigated the refinement of the RBD-1C aptamer against the SARS-CoV-2 S protein. Therefore, we aimed to fine tune the binding affinity of the RBD-1C aptamer through the proposed approaches. The mutated sequences were screened first based on their similarity in structure and on the basis of the docking score. MD was further applied to characterize the interaction of the selected aptamer with the S protein. The simulation and experimental results indicated that the selected DNA aptamer had better binding interactions with the S protein compared with its parent sequence and could be used as an alternative biological element for the detection of SARS-CoV-2 infection or as an antiviral candidate.

## 2. Results

### 2.1. Docking Simulation of the RBD-1C and Mutated Aptamers

**Figure 1:**
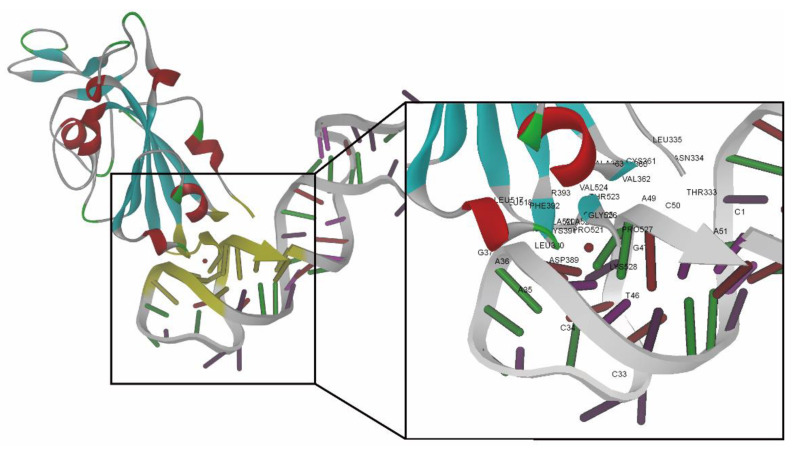
Best docking pose of RBD-1C aptamer against the S protein (visualization software: DS 4.1). Amino acids and nucleotides involved in the binding interface of the S protein/RBD-1C complex are marked with yellow. The close-up view presents the amino acids and nucleotides in the binding interface marked by labels.

**Figure 2:**
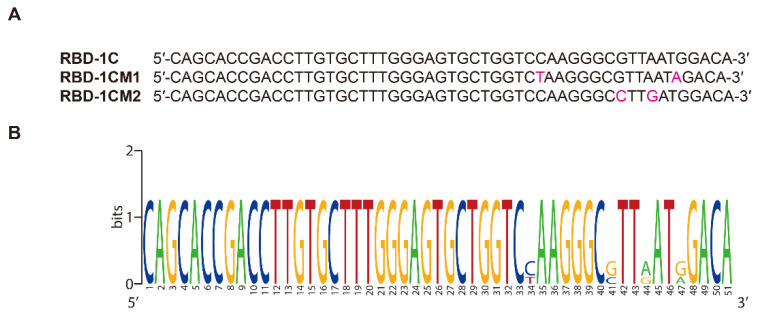
Sequence information of aptamers. (**A**) Sequences of the two best selected aptamers (RBD-1CM1 and RBD-1CM2) and RBD-1C aptamer. (**B**) Image of sequence alignment for three sequences (created by WebLogo) [29]. The mutated nucleotides are marked in this image.

The scores obtained by using the ZDOCK algorithm and ZRANK scoring function in the docking simulation were vital for the evaluation of intermolecular interactions. The ZDOCK and ZRANK scores for the best docking pose of the RBD-1C aptamer were 44.4 and −88.392, respectively. The docking result revealed that 24 of the 223 amino acids in the RBD of the S1 subunit were present in the binding interface between the aptamer and the S protein. These amino acids contain THR333, ASN334, LEU335, ASN360, CYS361, VAL362, ALA363, ASP389, LEU-390, CYS391, PHE392, THR393, LEU517, LEU518, HIS519, ALA520, PRO521, ALA-522, THR523, VAL524, CYS525, GLY-26, PRO527, and LYS-528 (Figure 1; nucleotides and amino acids listed in Table S1). The ZRANK score for the RBD-1C aptamer was used as a threshold value to screen the mutated sequences. A lower ZRANK score indicates improved docking prediction results, and 18 of the 595 mutated sequences screened by the Tanimoto similarity score had lower scores than the threshold value (Table S2 lists the detailed sequence information and docking results of the RBD-1C and 18 mutant aptamers). The two best selected aptamers were named as RBD-1CM1 and RBD-1CM2, which are potential aptamers for the S protein. Table 1 shows the molecular docking results of RBD-1C, RBD-1CM1, and RBD-1CM2 aptamers against the S protein. Figure S1 shows the secondary structures of RBD-1CM1 and RBD-1CM2 predicted by the RNAfold web server. Table 1 provides the initial number of hydrogen bonds present in the aptamer–protein complex. For the RBD-1CM1 aptamer, the mutated nucleotides were at positions 34 and 47 (Figure 2), and the ZRANK score for this aptamer was −98.551. The nucleotides and amino acids shown in the binding interface of the S protein/RBD-1CM1 complex (Table S1) differed from those of the other two complexes. A total of 38 amino acids (all in the RBD of S protein in the S1 subunit) and 16 nucleotides are involved in the binding interface. Figure S2 shows the best docking pose of the RBD-1CM1 aptamer against the S protein. The RBD-1CM2 aptamer has two mutated nucleotides at positions 41 and 44 in the sequence (Figure 2), and the docking result indicated that this aptamer is also a highly potential candidate for binding the S protein (ZRANK score: −97.133). In particular, the amino acids in the S protein and nucleotides in the RBD-1CM1 aptamer presented in the binding interface of the complex are the same as those in the best docking pose of the RBD-1C aptamer and the S protein (Table S1). Figure S3 shows the best docking pose of the RBD-1CM2 aptamer against the S protein. Hydrogen bonds are critical in the formation of aptamer–protein complexes. The VMD software predicted one, one, and two hydrogen bonds for tethering the S protein with the RBD-1C, RBD-1CM1, and RBD-1CM2 aptamers in the best docking poses, respectively.

### 2.2. MD Simulation of Protein–Aptamer Complexes

To better understand the interactions of protein–aptamer complexes, we used QwikMD [30] software to examine the efficacy interaction of the RBD-1C, RBD-1CM1, and RBD-1CM2 aptamers with the S protein. The structural stability of biomacromolecules was analyzed through MD simulation by identifying the following information: (i) the root-mean-square deviation (RMSD) from the initial structure of simulation, (ii) total energy, that is, the sum of potential and kinetic energies, and (iii) the number of hydrogen bonds (H-bonds).

#### 2.2.1. RMSD

**Figure 3:**
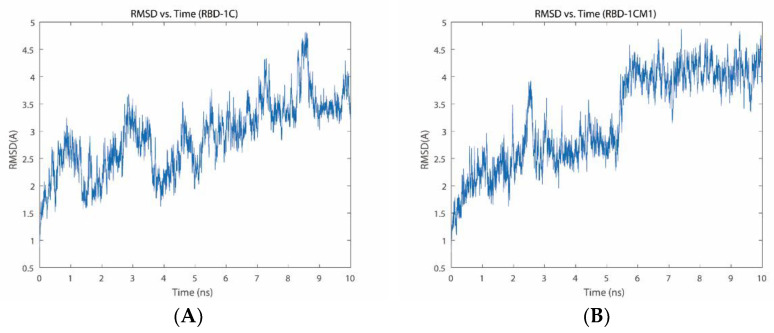

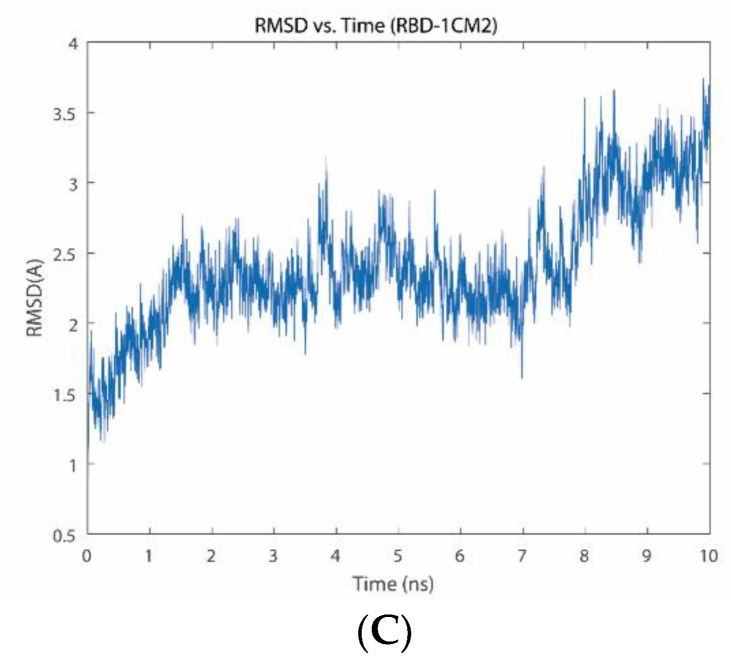
RMSD plots of the S protein/aptamer complexes during the MD simulation for (**A**) the S protein/RBD-1C complex, (**B**) S protein/RBD-1CM1 complex, and (**C**) S protein/RBD-1CM2 complex.

Figure 3 shows the RMSD plot of three aptamers, and the curves revealed several differences in the three aptamers during the simulation period. The highest RMSD value of the S protein/RBD-1C complex was 4.81 Å, and the average RMSD was 2.89 ± 0.65 Å. The fluctuations in the protein–aptamer interaction had an increasing trend from the onset to 7.5 ns of the simulation (Figure 3A). After 7.5 ns, the RMSD value showed a fluctuating trend but presented a tendency to stabilize. The S protein/RBD-1CM1 complex had the largest RMSD of 4.87 Å, with an average of 3.21 ± 0.86 Å. The complex reached a transient steady state after 3 ns in the simulation. However, a larger fluctuation appeared at 5.5 ns in the simulation. Afterward, an equilibrium state appeared from 6 ns to the end of the simulation. The highest RMSD value of the S protein/RBD-1CM2 complex was 3.74 Å, with an average of 2.40 ± 0.44 Å. The S protein/RBD-1CM2 complex could reach a short-term steady state from 2 ns to 7 ns in the simulation, but the RMSD increased steadily after 7 ns until the end of the simulation. Based on the RMSD values obtained from the 10 ns of the MD simulation, the RBD-1CM1 aptamer could be a superior recognition element for the S protein.

#### 2.2.2. Total Energy Analysis

**Figure 4:**
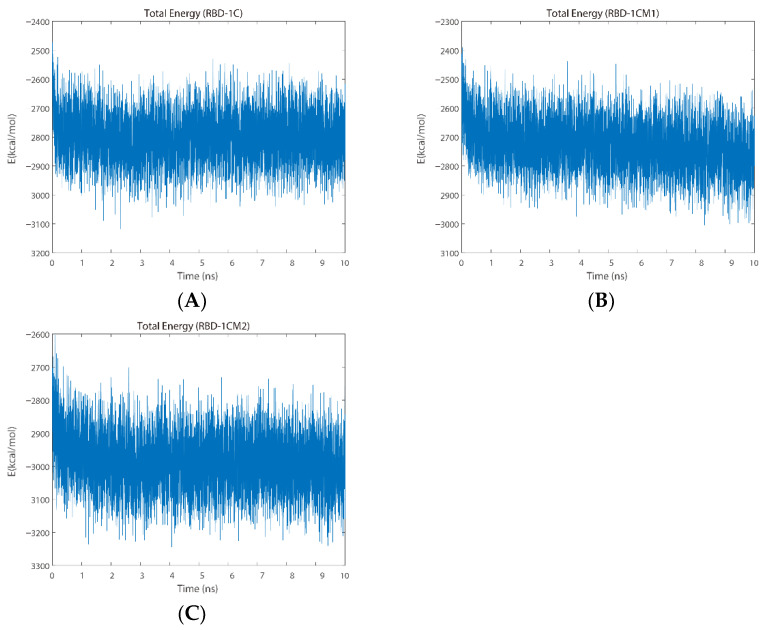
Total energy plots for (**A**) the S protein/RBD-1C complex, (**B**) S protein/RBD-1CM1 complex, and (**C**) S protein/RBD-1CM2 complex during the MD simulation.

Next, the total energy, including the potential and kinetic energies, was adopted to evaluate the binding between the aptamers and the S protein by using the function provided in QwikMD [30] software. The average total energy for the S protein/RBD-1C complex was −2801.9 ± 70.6 kcal/mol. The trajectories of the interaction between the RBD-1C aptamer and the S protein exhibited a slight decrease during the initial 2 ns of simulation and almost constant values until the end of the simulation (Figure 4A). The S protein/RBD-1CM1 complex had an average total energy of −2725.5 ± 72.7 kcal/mol, whereas its total energy slightly decreased at the end of the trajectory (Figure 4B). The RBD-1CM2 aptamer exhibited an average total energy of −2985.8 ± 71.1 kcal/mol in the trajectory of interacting with the S protein. From the total energy of simulation trajectories (Figure 4C), an evident decrease could be observed at the initial 2 ns of the simulation. The energy values remained stable in the subsequent simulation period. Specifically, the total energies for these three complexes were all negative and similar, which indicated that the complexes were stable systems. In summary, the data of total energy indicated the relative order of stable complexes during the simulation period: S protein/RBD-1CM2 complex > S protein/RBD-1C complex > S protein/RBD-1CM1 complex.

#### 2.2.3. Number of Hydrogen Bonds

**Figure 5:**
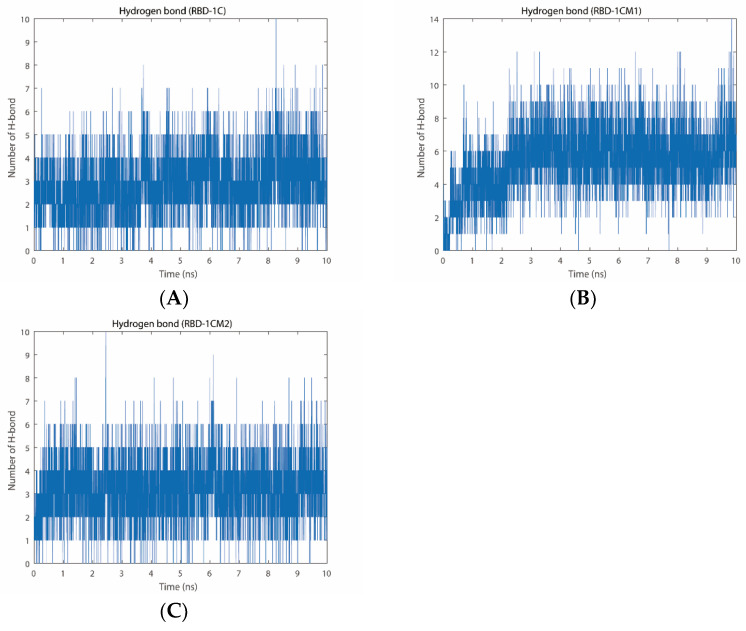
Number of hydrogen bonds of (**A**) the S protein/RBD-1C complex, (**B**) S protein/RBD-1CM1 complex, and (**C**) S protein/RBD-1CM2 complex during the 10 ns MD simulation.

**Figure 6:**
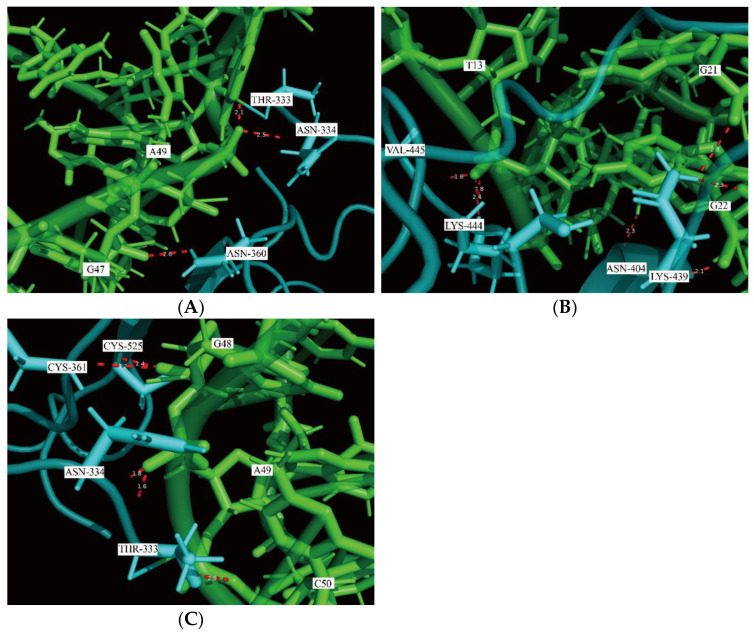
Visualization of the hydrogen bonds formed by the DNA aptamer and S protein in the last frame of MD simulation using PyMOL 2.5. The aptamer is colored in light green, the S protein in light blue, and hydrogen bonds shown by red dashed lines. (**A**) Three H-bonds formed in the binding interface of the S protein/RBD-1C complex, (**B**) seven H-bonds in the binding interface of the S protein/RBD-1CM1 complex, and (**C**) five H-bonds in the binding interface of the S protein/RBD-1CM2 complex.

The hydrogen bond is a type of polar binding between the interactions of molecular structures. Moreover, the number of hydrogen bonds can be used as a measure to evaluate the protein–aptamer interaction stability. Figure 5 shows the total number of hydrogen bonds that appeared in the trajectories of MD simulation for the RBD-1C, RBD-1CM1, and RBD-1CM2 aptamer against the S protein. The average number of hydrogen bonds was 2.89 ± 1.41 for the S protein/RBD-1C complex, with the highest number of 10 H-bonds and the lowest number of 0 H-bonds in the trajectory. The hydrogen bond disappearance in the trajectory occurred 140 times in 5000 simulation frames within 10 ns. During the simulation, the number of hydrogen bonds in the S protein/RBD-1C complex was nearly unchanged (Figure 5A). At 10 ns of simulation, the final number of hydrogen bonds between the S protein and RBD-1C aptamer was three. We used a molecular visualization software, PyMOL 2.5, to visualize the hydrogen bonds between the S protein and RBD-1C aptamer in the last frame of the MD simulation. Figure 6A shows the RBD-1C aptamer arranged with the hydrogen bonds with THR-333, ASN-334, and ASN-360 residues of the S protein through nucleotides A49 and G47.

The analysis of the interaction between the RBD-1CM1 aptamer and the S protein in the MD simulation showed that the average number of hydrogen bonds was 5.39 ± 2.07, with a maximum number of 14 hydrogen bonds. In particular, the number of hydrogen bonds in the binding interface of the S protein/RBD-1CM1 complex gradually increased at the initial 2.5 ns of simulation and subsequently reached a relatively stable state (Figure 5B), and the final number of H-bonds at 10 ns of simulation was seven. However, the hydrogen bond could also temporarily disappear in 36 of 5000 simulation frames within 10 ns. In the last frame of the MD simulation for the S protein/ RBD-1CM1 complex, hydrogen bonds appeared in ASN-439, ASN-440, LYS-444, and VAL-445 residues (Figure 6B). G21 and G22 nucleotides could form H-bonds with ASN-439, and the G22 nucleotide, especially, could have two H-bonds with ASN-440. In addition, T13 nucleotide equally made three stable interactions with LYS-444 and VAL-445 through H-bonds (Figure 6B). The S protein/RBD-1CM2 complex had an average number of hydrogen bonds of 3.15 ± 1.39, and the maximum number of hydrogen bonds was 10. Figure 5C shows that the number of hydrogen bonds remained stable, although with oscillation, during the simulation. Nevertheless, the situation of a temporarily missing hydrogen bond occurred 83 times in the 5000 frames of the simulation period. Importantly, the G48, A49, and C50 of the RBD-1M2 aptamer could form H-bonds with CYS-361 and CYS-525, THR-333 and ASN-334, and THR-333, respectively (Figure 6C).

Table S3 shows detailed information about the interactions and occupancy percentages of hydrogen bonds for the three complexes during the simulations. THR333, ASN334, ALA-522, and LYS528 were the most important amino acids to interact with the RBD-1C aptamer for the formation of hydrogen bonds. Only THR333 could have an occupancy rate for forming hydrogen bonds with the RBD-1C aptamer over 45% during simulation. The RBD-1CM1 aptamer could form hydrogen bonds with TYR369, THR376, LYS378, SER383, THR385, LYS386, ARG408, ASN440, LYS444, VAL445, and THR500, and the occupancy rates of these hydrogen bonds throughout the trajectory of the simulation were greater than 10%. For THR376, LYS378, ARG408, and LYS444, the occupancy rates were more than 45%. The RBD-1CM2 aptamer could interact with THR333, ASN334, ASN360, ALA522, and LYS528 to form hydrogen bonds with occupancy rates over 10% throughout the simulation. THR333 was the most important amino acid because its main structure and side chain could form hydrogen bonds with C50 and A49 of the RBD-1CM2 aptamer, with high occupancy rates (>45%). In light of the number of hydrogen bonds and the occurrence rate of zero hydrogen bonds, we suggested that the most stable complex was the S protein/RBD-1CM1 complex.

### 2.3. Quartz Crystal Microbalance (QCM) Experiments

**Figure 7:**
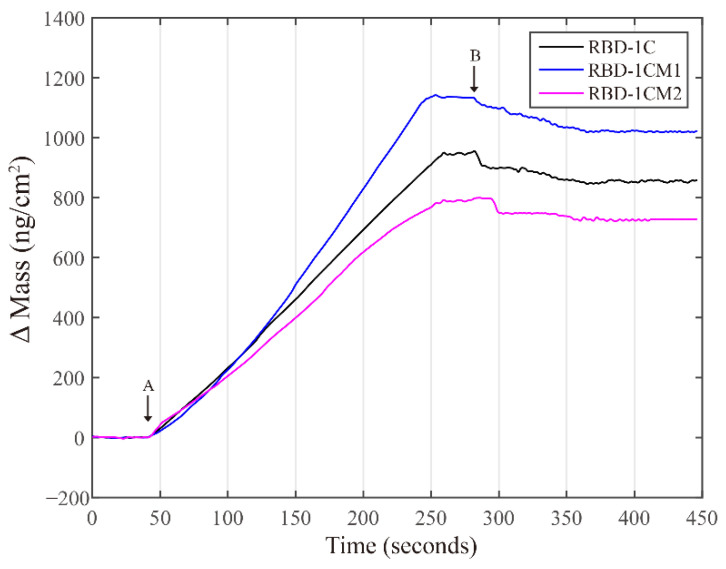
Representative time dependencies of Δ mass changes in the aptamer-immobilized chips respond-ing to the S protein. The S protein started to flow over the chip surface at arrow A (at 42 s) and the flow was changes to 1X PBS buffer at arrow B (at 282 s).

The QCM chip we used herein has a 7.995-MHz fundamental frequency, and a net change of 1 Hz corresponds to 6.837 ng/cm^2^ of mass change. The experiment for each aptamer was repeated three times. The frequency data were converted to the corresponding mass data, and the representative binding data are presented in Figure 7. After a stable baseline was achieved, the S protein was introduced to the flow channel at 42 s, and then the chip was rinsed with buffer at 282s, and the experiment was terminated at 446 s. Table 2 shows the average values of mass changes and kinetic parameters of aptamer–protein interactions. The data revealed that the RBD-1CM1 aptamer could have the biggest value in the mass change compared with the other two aptamers. From the viewpoint of binding affinity (*K*_*A*_), the values of RBC-1CM1 and RBC-1CM2 aptamers are equal, which are slightly bigger than that of the RBD-1C aptamer.

## 3. Discussion

The S protein on the surface of the SARS-CoV-2 virus can bind to ACE2 through the RBD of the S1 subunit. The RBD-1C aptamer can target the S protein through experiments [11,13] and simulations [13,14]. Given the intrinsic nature of nucleic acids, the DNA aptamer is relatively stable and easily undergoes chemical modification and immobilization and has low immunogenicity, less batch-to-batch variation, and high reproducibility [31,32]. Hence, the RBD-1C aptamer has a high potential to be used in the diagnosis, treatment, or prevention of SARS-CoV-2. In our study, we used computational approaches to select the mutated sequences generated based on the sequence of the RBD-1C aptamer. To the best of our knowledge, no other studies have reported aptamers for the SARS-CoV-2 S protein produced by using similar approaches. The 319–541 residues in the S1 subunit of the S protein constitute the RBD for the cell receptor ACE2 [3]; in addition, our simulation results confirm that the residues that interact with the RBD-1CM1 or RBD-1CM2 aptamer are all situated in the RBD of the S1 subunit. Compared with the RBD-1C aptamer, the RBD-1CM1 aptamer can form additional stable H-bonds with the S protein during the trajectory of simulation. Thus, the best aptamer for the SARS-CoV-2 S protein found in this study is the RBD-1CM1 aptamer.

In our study, we performed implicit solvent simulations, and the choice of solvent model was dictated by the speed of computation. However, the implicit solvent approach is less accurate than the explicit treatment of solvent in the simulation [33,34]. The implicit solvent models are still widely utilized in molecular simulations [35] because explicit computation is unnecessary for the extensive interactions between the atoms of individual solvent molecules, and implicit solvent simulations can sample conformational space faster and are suitable for binding affinity calculations [36], protein folding [37,38], and so forth. In the simulations of nucleic acids and protein–nucleic acid complexes using implicit solvent models, the results agree closely regarding conformational sampling from explicit solvent simulations or experimental data [39]. In addition, the implicit solvent model used in the simulation can offer rational agreement with the observables from the experiment in the prediction of effects of protein phosphorylation [40].

Several researchers adopted a genetic algorithm based on the in-silico maturation strategy to design new aptamers from a known aptamer [41,42]. By using their proposed methods, the affinity and selectivity of oligonucleotide aptamers can be improved in comparison with the parent aptamer. In our study, the generation of new sequences from a known aptamer for computational selection was performed in accordance with the mutation rules. The screening procedures of structure similarity, docking, and MD simulations can be applied to perform the in-silico selection of aptamers for screening a large number of mutated sequences. The computational results obtained from our study reveal that the RBD-1CM1 aptamer can have strong interactions with the RBD of the S1 subunit, which also has the potential for diagnosis applications, such as in rapid test kits [12] and aptamer-based biosensors [11], or for the treatment of SARS-CoV-2 [43].

The experimental data reveal that the RBC-1CM1 and RBC-1CM2 aptamers improved binding affinity to the S protein. However, the effect of increasing the binding affinity of the two aptamers is slight. From the viewpoint of improving binding affinity, the experimental results are in line with the results predicted by the simulations. However, the experimental results do not exhibit great significant improvements as predicted by the simulations. Hence, experiments are indispensable for verifying the real performance of the selected candidates from computational studies. Even so, the experimental data support that the refinement of the binding affinity of the aptamer against the S protein through the computational methods proposed in this study is feasible. In addition, it is worth noting that the average final mass change for the RBC-1CM2 aptamer is the smallest among the three aptamers. Hence, the experimental data support that the RBC-1CM1 aptamer is the best aptamer among the three aptamers, considering the values of binding affinity and mass change.

Herein, we performed 595 repetitions of aptamer–protein docking simulations to select a mutated sequence generated from the RBD-1C aptamer with a better binding capability against the S protein. The MD simulation was also applied to characterize the binding interactions of RBD-1C and two selected aptamers with the S protein. Based on these results, the RBD-1CM1 aptamer is the best aptamer, presenting better docking and MD simulation results, improved binding affinity, and higher response signals of QCM measurements. The best selected aptamer can be an ideal recognition probe for SARS-CoV-2 RBD because the MD simulations validate that the RBD-1CM1 aptamer can form more hydrogen bonds with the S protein compared with the RBD-1C and RBD-1CM2 aptamers. These in-silico analysis findings and experimental data support that the RBD-1CM1 aptamer is superior to the RBD-1C aptamer. In practice, the obtained aptamer can be an alternative to antibodies and has promising applications, such as aptamer-based biosensors or LFAs, in the treatment and diagnosis of SARS-CoV-2. The limitations of the present study in the simulations are that only the RBD of S protein is adopted, and the simulation time of MD is limited by the computing power. Further experiments and simulations will be conducted for validating the characteristics of other selected aptamers (shown in Table S2) in our future studies.

## 4. Material and Methods

**Figure 8:**
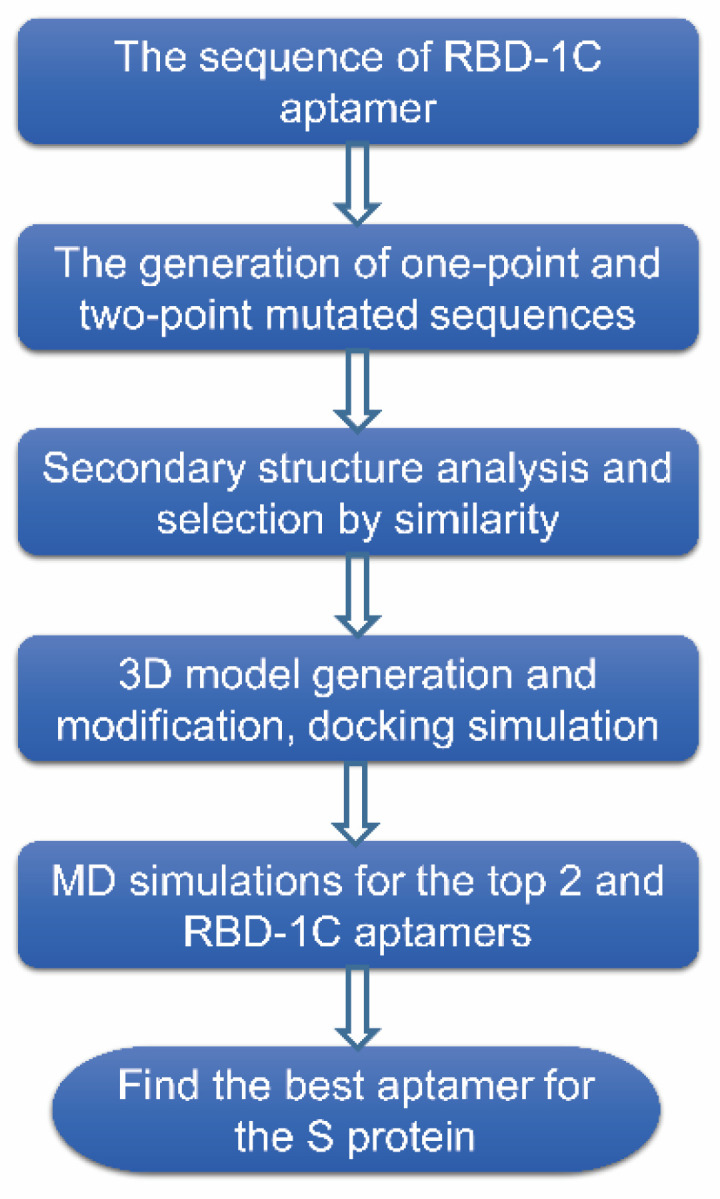
Schematic showing the workflow for the in-silico selection of aptamer target SARS-CoV-2 S protein.

In this study, we adopted computational approaches to carry out the in-silico selection of aptamer target SARS-CoV-2 S protein. Figure 8 schematizes the workflow for the selection of the DNA aptamer against S protein, and the best aptamer was predicted to have a better binding capability compared with the original (parent) aptamer sequence based on the simulation results. The detailed steps for the workflow are described in the following paragraphs. In addition, a QCM was utilized to test the performance of two selected and RBD-1C aptamers.

### 4.1. Sources of Protein and Aptamer Data

**Figure 9:**
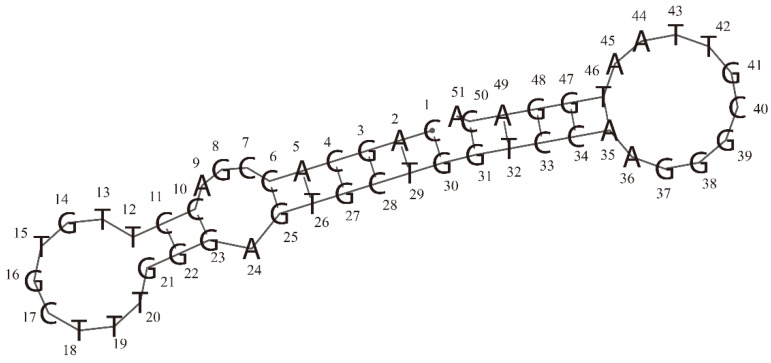
Secondary structure of the RBD-1C aptamer was predicted by the RNAfold web server [45]. In this structure, 2 hairpins (at two ends) and 1 internal loop can be observed.

The crystal structure of the S protein of SARS-CoV-2 was acquired from Protein Data Bank. This file (PDB ID: 7DPM) contains the S protein of SARS-CoV-2 and a fully human cross-reactive neutralizing monoclonal antibody (MW06), which could bind the spike RBDs of SARS-CoV-2 and SARS-CoV [44]. To prepare the crystal structure of the S protein for simulation, we cleaned this file and removed the unwanted molecules. The sequences of aptamers that could bind SARS-CoV-2 RBD were acquired from a previous report [13]. These sequences (CoV2-RBD-1 and CoV2-RBD-4) had K_d_ values of 5.7 and 19.9 nM, respectively. We finally selected the CoV2-RBD-1 (or RBD-1C) aptamer as the parent sequence for the generation of mutated sequences used in the in-silico study. The RBD-1C aptamer consists of 51 DNA nucleotides, yielding 2 hairpins and 1 internal loop in its secondary structure (as shown in Figure 9; predicted by the RNAfold web server [45]; 5′-CAGCACCGACCTTGTGCTTTGGGATGCTGGTCCAAGG GCGTTAATGGACA -3′).

### 4.2. Generation of Mutated Sequences

To trigger the maturity of the binding capability of RBD-1C aptamer against SARS-CoV-2 RBD, we retrieved the two fragments (3rd to 28th and 32nd to 49th) from the sequence of RBD-1C to explore appropriate methods for the generation of mutant sequences. Figure S4 shows the predicted secondary structures of two fragments. The figure reveals that the secondary structure of these fragments contained two parts of secondary structures in intact sequence. Next, the RNAfold web server was utilized to analyze the sequences, and tertiary structures of aptamers were generated using RNAComposer [46]. The ZDOCK docking program was applied to dock the two three-dimensional (3D) models of aptamers individually with the S protein model. To the best of our knowledge, ZDOCK is one of the preferred docking algorithms to investigate the binding interaction in the aptamer–ligand complex [47]. The ZDOCK employs the fast Fourier transform algorithm to perform the rigid-body docking (with 6° angular sampling) in a global search manner and measures the degree of shape complementarity, electrostatics, and statistical potential terms for scoring.

The preliminary simulation result revealed that the ZDOCK score for the first fragment (the 3rd to 28th) was 42.12, and that of the second fragment (the 32nd to 49th) was 44.53. Owing to the short lengths of these fragments, the evaluation of binding capability relied on the ZDOCK score. The docking result revealed the importance of the second fragment (32nd to 49th) in binding the S protein of SARS-CoV-2. Therefore, we decided to produce one-point and two-point mutated sequences starting from the 32nd to the 49th nucleotide in the sequence of the RBD-1C aptamer. We suggested that the minor changes in the nucleotides of RBD-1C aptamer may increase its binding capability toward the S protein.

Initially, 54 one-point mutated sequences were produced by replacing the nucleotides at positions 32 to 49. For example, the original nucleotide at position 32 was T and it could be replaced with A, G, or C. Therefore, three mutated sequences could be obtained at each position. The two-point mutations started from positions 32 and 33 and then mutated at positions 32 and 34, and the procedure was progressively followed until positions 32 and 49 were reached. Afterward, the next round of mutations started from positions 33 and 34 and ended at positions 33 and 49. Following the rule for generating two-point mutated sequences, this procedure was repeated until positions 48 and 49 were reached. Finally, 1377 two-point mutated sequences were generated. The total number of mutated sequences, including one-point and two-point mutated sequences generated in this study, was 1431.

### 4.3. Comparison of Similarity in the Secondary Structure

The secondary structure of each mutated sequence was analyzed by using the RNAfold web server [45], and the dot-bracket notation was recorded in an Excel file. For comparison of the structural similarity of the mutated sequence with the RBD-1C aptamer, the string format of the dot-bracket representation was used in the calculation of the similarity of two sequences. We used the Tanimoto similarity score [48] to calculate the coefficient of similarity of two strings with the same length. The equation for the Tanimoto similarity score is as follows:

Tanimoto similarity score = NIdent/NSum,(1)

where NIdent means the number of matching positions in the string format of dot-bracket representation and NSum is the length of the string. The Tanimoto similarity score ranges from 0 to 1. We wrote a C# program to calculate the Tanimoto similarity score and set a cutoff value of 0.96 to screen the mutated sequences. When the Tanimoto similarity score of a sequence was below 0.96, it was excluded in the subsequent docking simulation. After using this exclusion criterion, 595 out of 1431 mutated sequences passed the threshold and were adopted in the subsequent ZDOCK simulations.

### 4.4. Generation of the 3D Aptamer Structure and Molecular Simulations

The tertiary structure of aptamer was then generated with the use of RNAComposer via inputting the necessary sequence and secondary structure information in dot-bracket format. After obtaining the 3D model of aptamer, the nucleotides in the RNA structure were modified by using Accelrys Discovery Studio (DS) 4.1 (currently BIOVIA Discovery Studio) to replace uracil (U) with thymine (T) base. The models of DNA aptamers were used in the subsequent molecular simulations. The ZDOCK docking program combined with the ZRANK scoring function in the DS software was utilized to assess the interactions between the aptamer and the target protein molecule. According to a previous finding [22], the experimental results were more consistent with the simulation findings when using the ZRANK score in the evaluations of interactions of aptamers with long sequences with the protein. For short sequences, the ZDOCK score can provide accurate prediction results for the aptamer–protein interaction [26]. The CoV2-RBD-1 aptamer is a long-sequence aptamer with a length that reaches 51 nucleotides. Hence, the ZRANK score was adopted to evaluate the binding capability of aptamers to the S protein. The reranking program, ZRANK, adopted detailed electrostatics, van der Waals, and desolvation in the scoring function, which improved the success rate over the ZDOCK predictions [49]. The MD simulation was run based on the best configuration of the protein–aptamer complex obtained from the ZDOCK prediction. QwikMD [30], incorporating commonly used functions of NAMD [50] and VMD [51,52] and offering the graphic user interface for MD simulations, was utilized in this study. The MD simulations were carried out under the implicit solvation model with a salt concentration of 0.15 mol/L at a temperature of 27 °C. The total time of each MD simulation was 10 ns. We also adopted PyMOL 2.5 (Schrödinger, Inc., New York, NY, USA) software to visualize molecules. In this study, all computations were carried out on the HP Z620 desktop workstation, which contains 24 computing cores, 44 GB RAM, 1 NVIDIA Quadro K600, and a 64-bit Window 10 operating system.

### 4.5. Experimental Section

Phosphate-buffered saline (PBS) (10X) was purchased from Thermo Scientific (Waltham, MA, USA) and diluted to 1X PBS (adjusted pH to 7.4 with the HCl solution) for preparing protein and aptamer solutions at specified concentrations. Polyethylene glycol (PEG) thiols (silane-PEG-NH_2_, 1K and silane-PEG-OH, 1K) were acquired from Biochempeg Scientific Inc. (Watertown, MA, USA). Aptamers with the 5′-amino-modifier C6 modification were synthesized by MDBio Inc. (New Taipei City, Taiwan), and the recombinant SARS-CoV-2 spike protein (S1 subunit) was obtained from RayBiotech Life, Inc. (Peachtree Corners, GA, USA). Glutaraldehyde, sodium cyanoborohydride (NaBH_3_CN), and tris(hydroxymethyl)aminomethane hydrochloride (Tris-HCl) were all purchased from Sigma-Aldrich (Burlington, MA, USA). Other chemicals used in this study were all reagent grade. The experiments were carried out on an electrochemical QCM (EQCM) workstation (CHI 410C, CH Instruments, Inc., Austin, TX, USA).

Before functionalizing the surface of the QCM chip, a UV/ozone cleaner was used to irradiate the chip for 5 min in order to remove contaminants. After that, the chip was immersed in the solution of mixed PEG thiols (PEG-NH_2_ (10 mg) and PEG-OH (10 mg) dissolved in 2 mL of 99.9% ethanol) at room temperature. After overnight reaction, the chip was rinsed with ethanol and blown dry with nitrogen. The mixed self-assembled monolayer (mSAM) on the chip was then functionalized with glutaraldehyde (2.5% in deionized (DI) water) for 60 min, and the chip was subsequently cleaned with DI water. Afterward, the chip was incubated in the solution containing 0.1 μM aptamer at room temperature overnight. After rinsing the chip with DI water, the chip was incubated in the Tris-HCl solution (10 mM) containing 4% NaBH_3_CN for 30 min to block unreacted active functional groups. Figure S5A presents the schematic diagram illustrating the chemical modifications on the chip surface. The QCM flow cell (shown in Figure S5B) and a peristaltic pump constituted the flow channel system to transport liquid flowing through the chip surface. The equations used to calculate the kinetic parameters of reactions are presented in the Experimental Section of Supplementary Material.

## Figures and Tables

**Table 1:** Docking results of RBD-1C, RBD-1CM1, and RBD-1CM2 aptamer against the S protein. The number of hydrogen bonds in the interface of the best docking pose of the complex was predicted by using the VMD software.

ID	ZDOCK Score	ZRANK Score	The Number of Hydrogen Bonds
RBD-1C	44.4	−88.392	1
RBD-1CM1	51.5	−98.551	1
RBD-1CM2	44.7	−97.133	2

**Table 2:** Results of QCM experiments for RBD-1C, RBD-1CM1, and RBD-1CM2 aptamer against the S protein. Under the experimental conditions, the average values of mass changes and kinetic parameters are presented as follows. *k_a_*: association constant; *k_d_*: dissociation constant; *K_A_* = *k_a_*/*k_d_* (binding affinity).

ID	Biggest Δmass (ng/cm^2^)	Final Δmass (ng/cm^2^)	*K*_*a*_ (×10^3^ M^−1^s^−1^)	*K*_*d*_ (×10^−3^ s^−1^)	*K*_*A*_ (×10^5^ M^−1^)
RBD-1C	948 ± 23.7	847.6 ± 42.2	0.118	1.193	1.0
RBD-1CM1	1065 ± 38.6	964.2 ± 51.4	0.124	1.029	1.2
RBD-1CM2	812.8 ± 34.3	727.8 ± 46.5	0.125	1.020	1.2

## Data Availability

The data that support the findings of this study are available from the corresponding author on reasonable request.

## Supplementary Materials

The following are available online at https://www.mdpi.com/article/10.3390/ijms23105810/s1.
