# Supplementary material for: In-Silico Selection of Aptamer Targeting SARS-CoV-2 Spike Protein
Source: Int J Mol Sci. 2022 May 22;23(10):5810. doi: 10.3390/ijms23105810 (PMC9143595; doi:10.3390/ijms23105810)
Supplement: Supplementary file 1 [file ijms-23-05810-s001.zip › ijms-1506567-supplementary.pdf]

Supplementary material

In-Silico Selection of Aptamer Targeting SARS-CoV-2 Spike Protein

Yu-Chao Lin [1,2,†], Wen-Yih Chen [3,†], En-Te Hwu [4] and Wen-Pin Hu [5,6,*]

[1] Division of Pulmonary and Critical Care Medicine, Department of Internal Medicine, China Medical University Hospital, Taichung 404333, Taiwan; d10001@mail.cmuh.org.tw
[2] School of Medicine, China Medical University, Taichung 404333, Taiwan
[3] Department of Chemical and Materials Engineering, National Central University, Jhong-Li 32001, Taiwan; wychen@ncu.edu.tw
[4] Department of Health Technology, Technical University of Denmark, 2800 Lyngby, Denmark; etehw@dtu.dk
[5] Department of Bioinformatics and Medical Engineering, Asia University, Taichung 41354, Taiwan
[6] Department of Medical Research, China Medical University Hospital, China Medical University, Taichung 40447, Taiwan
* Correspondence: wenpinhu@asia.edu.tw
† These authors contributed equally to this work.

Experimental section

After getting experimental frequency data, the data are converted to the corresponding data, and a net change of 1 HZ corresponds to 6.837 ng/cm^2 of mass change. Initially, the data are divided into four sections: baseline, association, dissociation, and equilibrium. For getting the dissociatation constant, Eq. 1. was utilized to fit the data of dissociation segment.

$$M_t = M_0 e^{-k_d(t-t_0)} \quad (1)$$

Based on the equation reported in the literature [1], Eq. 1 is derived by changing the frequence to mass. Here, M_t denotes the change in mass at time t in ng/cm^2, t_0 represents the starting time of dissociation, M_0 denotes the value of mass change at the starting time, and k_d is the dissociation constant.

For fitting the association segment of data, the binding between the probe and the target molecules is described by Eqs. 2-4 [2].

$$[\text{target}] + [\text{probe}] \underset{k_d}{\overset{k_a}{\rightleftarrows}} [complex] \quad (2)$$

$$\Delta M_t = \Delta M_{max}\{1 - \exp[-(\frac{1}{\tau})]\} \quad (3)$$

$$\tau^{-1} = k_a + k_d \quad (4)$$

where ΔM_t denotes the change in mass from the start of the association to time t, and ΔM_{max} is the mass shift at the plateau of the sensorgram. The binding constant, K_A, can be calculated by using Eq. 5.

$$K_A = \frac{k_a}{k_d} \quad (5)$$

The kinetic parameters are calculated by using Matlab 2020b (The MathWorks, Inc., USA), and the data are imported with the use of the script. Then, the curve fitting toolbox (cftool) is utilized to fit the data by giving Eq. 1 or 3 for getting the kinetic parameters.

References

1. Heller, G.T.; Mercer-Smith, A.R.; Johal, M.S.Quartz Microbalance Technology for Probing Biomolecular Interactions BT - Protein-Protein Interactions: Methods and Applications. In; Meyerkord, C.L., Fu, H., Eds.; Springer New York: New York, NY, 2015; pp. 153–164 ISBN 978-1-4939-2425-7.
2. OKAHATA, Y.; NIIKURA, K.; FURUSAWA, H.; MATSUNO, H.A Highly Sensitive 27 MHz Quartz-Crystal Microbalance as a Device for Kinetic Measurements of Molecular Recognition on DNA Strands. *Anal. Sci.* **2000**, *16*, 1113–1119, doi:10.2116/analsci.16.1113.

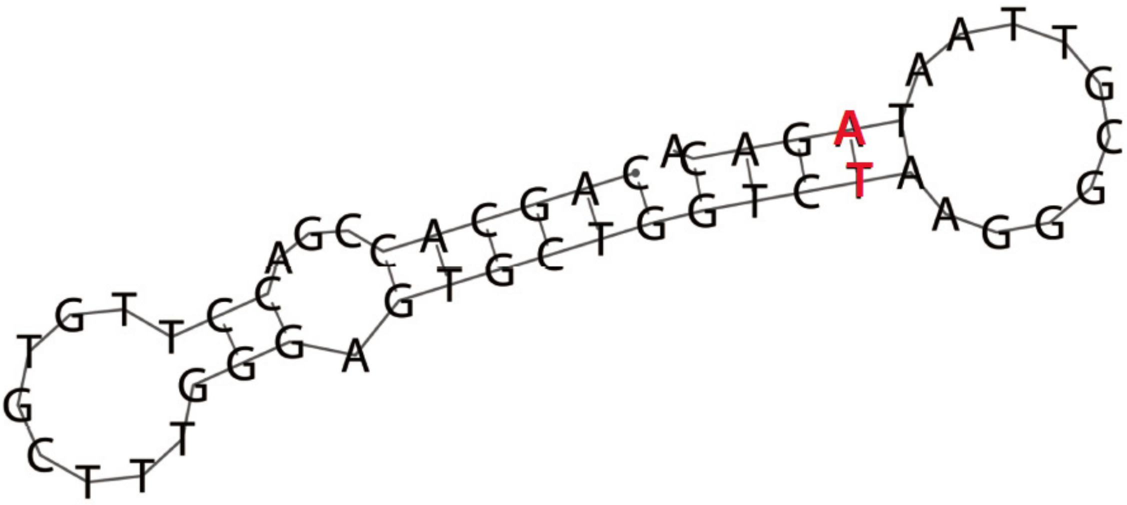

(A)

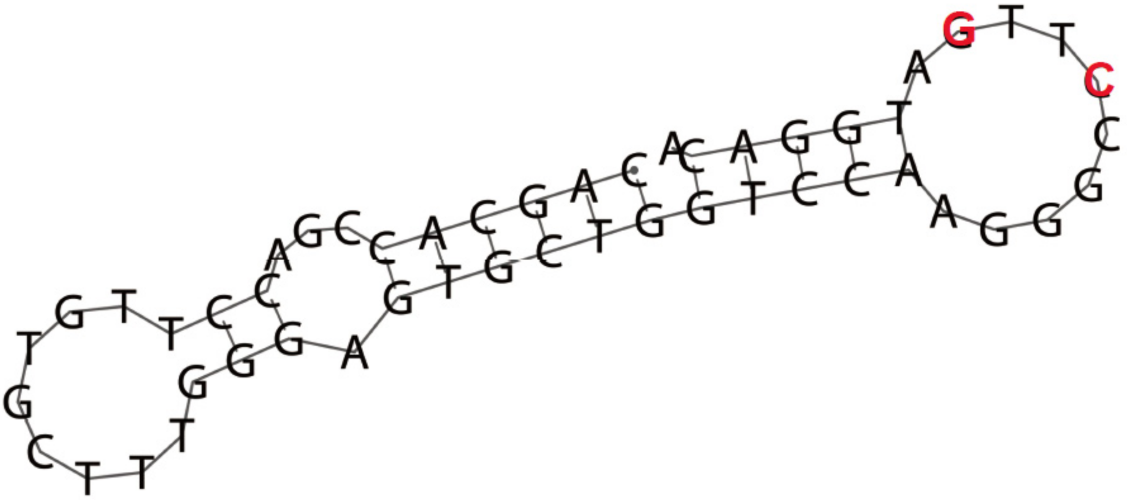

(B)

Figure S1. The secondary structures of mutant aptamers predicted by RNAfold web server. (A) RBD-1CM1 (B) RBD-1CM2. The mutant nucleotides are marked in red.

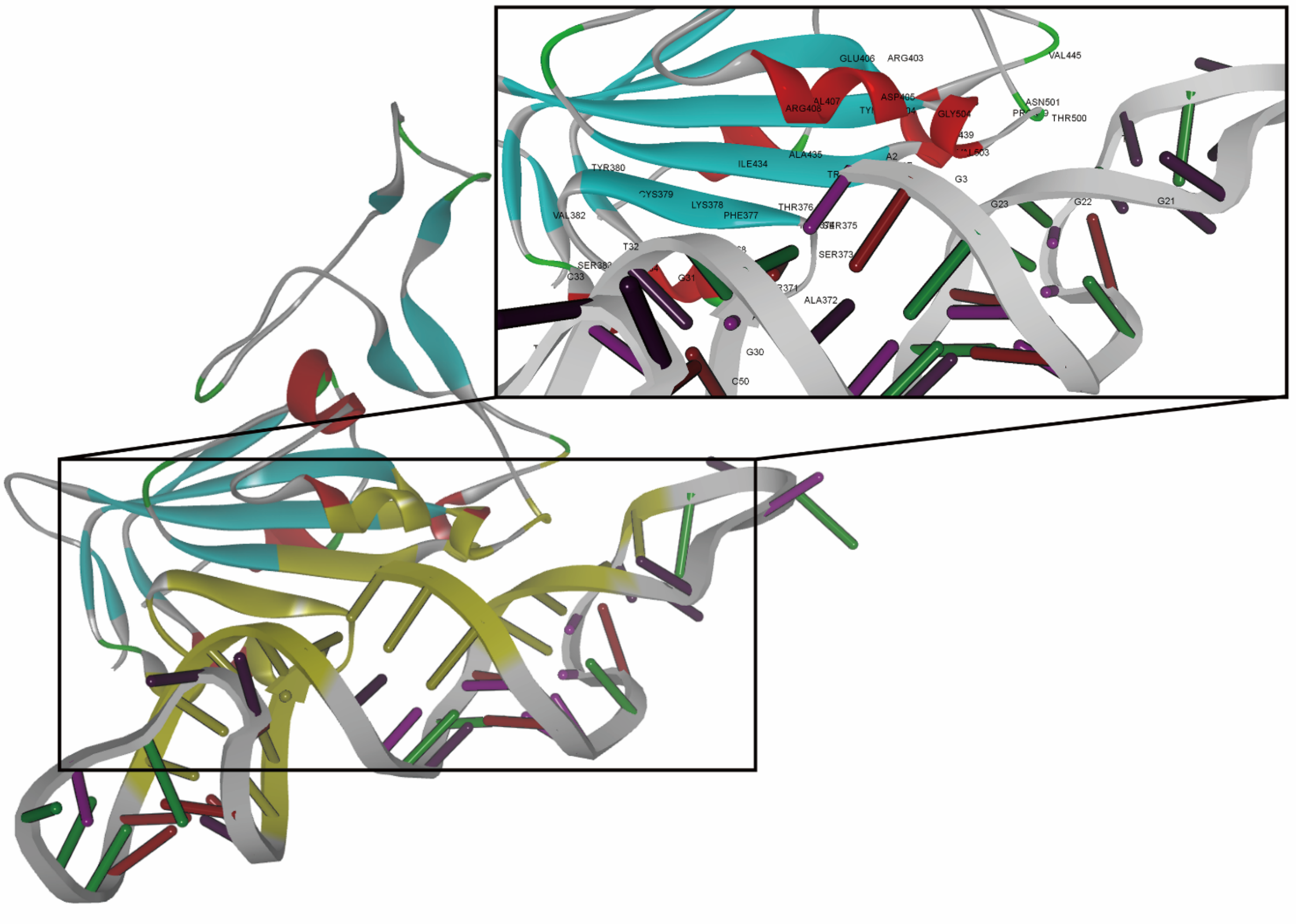

Figure S2. Best docking pose of RBD-1CM1 aptamer against the S protein. The amino acids and nucleotides involved in the binding interface of the S protein/RBD-1CM1 complex are marked with yellow. In this binding interface, it contains 38 amino acids (all in the RBD of S protein in the S1 subunit) and 16 nucleotides (information shown in Table S1).

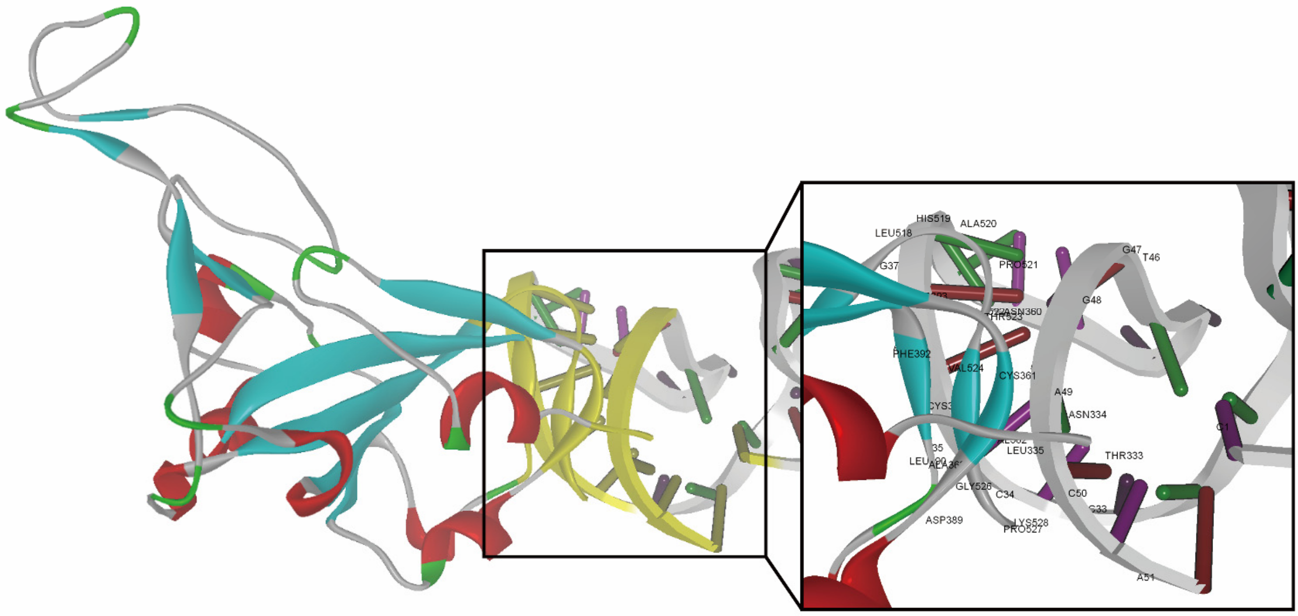

Figure S3. Best docking pose of RBD-1CM2 aptamer against the S protein. The amino acids and nucleotides involved in the binding interface of the S protein/RBD-1CM2 complex are marked with yellow. The amino acids and nucleotides present in the binding interface are the same as those shown in the binding interface of the S protein/RBD-1C complex (information shown in Table S1).

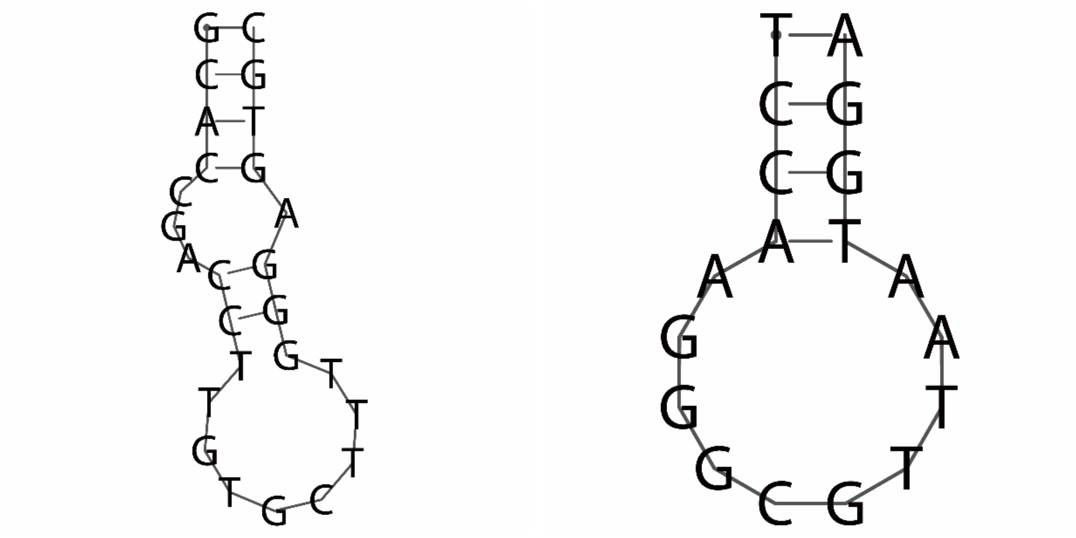

(A) (B)

Figure S4. The two fragments originate from the sequence of RBD-1C aptamer. (A) The predicted secondary structure comprises the 3rd to 28th nucleotides of the RBD-1C aptamer. (B) The predicted secondary structure comprises the 32nd to 49th nucleotides of the RBD-1C aptamer.

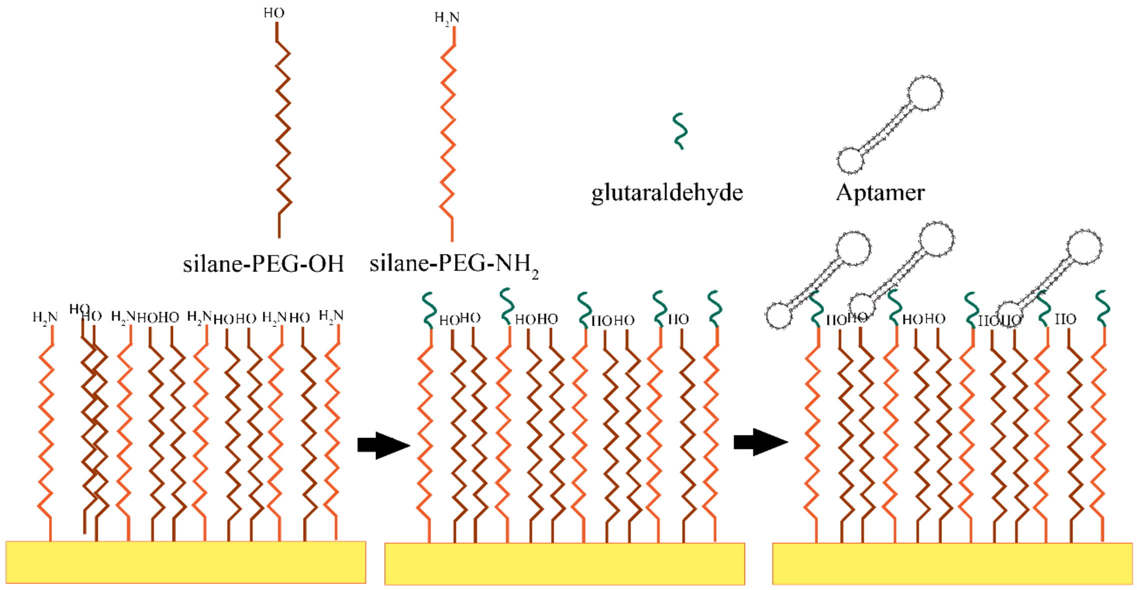

(A)

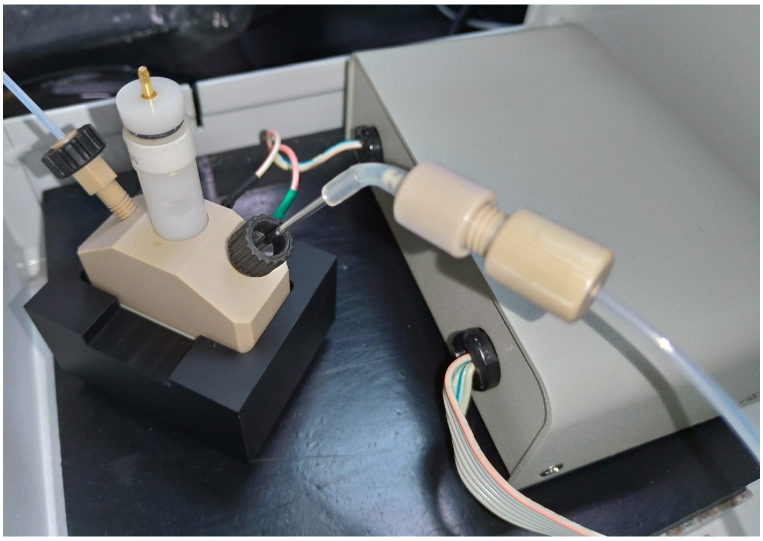

(B)

Figure S5. The steps of functionalizing the chip surface, and the setup of the QCM flow cell. (A) The surface of the QCM chip is modified with the mixed self-assembled monolayer (mSAM) of silane-PEG (silane-PEG-NH_2:silane-PEG-OH = 1:1) and glutaraldehyde before the immobilization of aptamer. (B) The setup of the QCM flow cell.

Table S1. Amino acids and nucleotides involved in the binding interfaces of complexes.

Binding interface of S protein/RBD-1C complex		Binding interface of S protein/RBD-1CM1 complex		Binding interface of S protein/RBD-1CM2 complex	
**Amino acid**	**nucleotide**	**Amino acid**	**nucleotide**	**Amino acid**	**nucleotide**
THR333	C1	LEU368	C1	THR333	C1
ASN334	C33	TYR369	A2	ASN334	C33
LEU335	C34	ASN370	G3	LEU335	C34
ASN360	A35	SER371	T13	ASN360	A35
CYS361	A36	ALA372	G21	CYS361	A36
VAL362	G37	SER373	G22	VAL362	G37
ALA363	T46	PHE374	G23	ALA363	T46
ASP389	G47	SER375	G30	ASP389	G47
LEU390	G48	THR376	G31	LEU390	G48
CYS391	A49	PHE377	T32	CYS391	A49
PHE392	C50	LYS378	C33	PHE392	C50
THR393	A51	CYS379	T34	THR393	A51
LEU517		TYR380	G48	LEU517	
LEU518		VAL382	A49	LEU518	
HIS519		SER383	C50	HIS519	
ALA520		PRO384	A51	ALA520	
PRO521		THR385		PRO521	
ALA522		LYS386		ALA522	
THR523		ARG403		THR523	
VAL524		GLY404		VAL524	
CYS525		ASP405		CYS525	
GLY526		GLU406		GLY526	
PRO527		VAL407		PRO527	
LYS528		ARG408		LYS528	
		ILE434			
		ALA435			
		TRP436			
		ASN437			
		ASN439			
		ASN440			
		VAL445			
		PRO499			
		THR500			
		ASN501			
		VAL503			

Table S1 (continued).

		GLY504			
		GLN506			
		TYR508			

Table S2. Sequence information and docking results of the RBD-1C and top 18 aptamers. The red upper letters in sequences indicate the locations of mutated nucleotides.

Name	Sequence(5'→ 3')	ZRANK score
**RBD-1CM1**	CAGCACCGACCTTGTGCTTTGGGAGTGCTGGTCTAAGGGCGTTAATAGACA	-98.551
**RBD-1CM2**	CAGCACCGACCTTGTGCTTTGGGAGTGCTGGTCCAAGGGCCTTGATGGACA	-97.133
**RBD-1CM3**	CAGCACCGACCTTGTGCTTTGGGAGTGCTGGTCCAAGAGCGTTAGTGGACA	-95.996
**RBD-1CM4**	CAGCACCGACCTTGTGCTTTGGGAGTGCTGGTCCAACGGCGTTACTGGACA	-95.524
**RBD-1CM5**	CAGCACCGACCTTGTGCTTTGGGAGTGCTGGTCCAAGGTCGTTAACGGACA	-95.380
**RBD-1CM6**	CAGCACCGACCTTGTGCTTTGGGAGTGCTGGTCCAAGGGCCATAATGGACA	-94.235
**RBD-1CM7**	CAGCACCGACCTTGTGCTTTGGGAGTGCTGGTCCAAGGGCGTTCACGGACA	-92.578
**RBD-1CM8**	CAGCACCGACCTTGTGCTTTGGGAGTGCTGGTCCAAGGGCTTTATTGGACA	-92.37
**RBD-1CM9**	CAGCACCGACCTTGTGCTTTGGGAGTGCTGGGCCAAGGGCGTTAATGGCCA	-92.225
**RBD-1CM10**	CAGCACCGACCTTGTGCTTTGGGAGTGCTGGTCCAAAGTCGTTAATGGACA	-91.376
**RBD-1CM11**	CAGCACCGACCTTGTGCTTTGGGAGTGCTGGTCCAACGGGGTTAATGGACA	-90.607
**RBD-1CM12**	CAGCACCGACCTTGTGCTTTGGGAGTGCTGGTCCAAGGGTCTTAATGGACA	-90.550
**RBD-1CM13**	CAGCACCGACCTTGTGCTTTGGGAGTGCTGGTCCAAGACCGTTAATGGACA	-90.326
**RBD-1CM14**	CAGCACCGACCTTGTGCTTTGGGAGTGCTGGTCCACGGGCGTCAATGGACA	-90.113
**RBD-1CM15**	CAGCACCGACCTTGTGCTTTGGGAGTGCTGGTCCTAGGGCGTTAACGGACA	-89.35
**RBD-1CM16**	CAGCACCGACCTTGTGCTTTGGGAGTGCTGGTCCAAGTGCGTTAGTGGACA	-88.947
**RBD-1CM17**	CAGCACCGACCTTGTGCTTTGGGAGTGCTGGTCCAGAGGCGTTAATGGACA	-88.556
**RBD-1CM18**	CAGCACCGACCTTGTGCTTTGGGAGTGCTGGTCCAAGGACGGTAATGGACA	-88.533
**RBD-1C**	CAGCACCGACCTTGTGCTTTGGGAGTGCTGGTCCAAGGGCGTTAATGGACA	**-88.392**

Table S3. The MD results of the hydrogen bonding interactions for the three complexes show a percentage greater than one-tenth of the trajectory (1/10 of 5000 frames).

ID_Complex	Donor	Acceptor	Occupancy (%)
**S protein/RBD-1C**	THR333-Main	C50-Side	51.61
	THR333-Side	C50-Side	43.91
	LYS528-Side	C33-Side	27.21
	G47-Side	ALA522-Main	24.40
	THR333-Main	A51-Side	23.28
	LYS528-Side	A49-Side	21.00
	THR333-Main	A49-Side	19.30
	ASN334-Main	A49-Side	17.24
	LYS528-Side	C50-Side	11.88
**S protein/RBD-1CM1**	THR376-Side	A2-Side	65.9
	ARG408-Side	G3-Side	62.26
	LYS378-Side	A2-Side	46.38
	ARG408-Side	A2-Side	46.34
	LYS444-Side	T12-Side	45.56
	LYS444-Side	T13-Side	38.9
	VAL445-Main	T13-Side	29.3
	TYR369-Side	G31-Side	22.18
	LYS386-Side	C33-Side	16.8
	THR500-Side	T19-Side	16.68
	G23-Side	ASN440-Side	14.64
	THR385-Side	C33-Side	12.72
	SER383-Side	T32-Side	12.48
**S protein/RBD-1CM2**	THR333-Main	C50-Side	63.8
	THR333-Side	C50-Side	47.74
	THR333-Main	A49-Side	46.08
	T47-Side	ALA522-Main	33.30
	ASN334-Main	A49-Side	27.56
	LYS528-Side	C50-Side	25.7
	ASN334-Side	A49-Side	19.98
	ASN360-Side	G47-Side	14.24
	LYS528-Side	A49-Side	12.32
